# Improved ROSC rates in out-of-hospital cardiac arrest patients after introduction of a text message alert system for trained volunteers

**DOI:** 10.1007/s12471-021-01656-6

**Published:** 2022-01-06

**Authors:** D. M. Oosterveer, M. de Visser, C. Heringhaus

**Affiliations:** 1 Basalt Rehabilitation Centre, Leiden/The Hague, The Netherlands; 2 Department of Research and Development, Hollands Midden Regional Ambulance Service, Leiden, The Netherlands; 3 Emergency Department, Leiden University Medical Centre, Leiden, The Netherlands

**Keywords:** Cardiac arrest, Bystander cardiopulmonary resuscitation, Utstein template, Resuscitation, Survival

## Abstract

**Objective:**

To evaluate whether a text message (TM) alert system for trained volunteers contributed to early cardiopulmonary resuscitation, the use of automated external defibrillators (AEDs), return of spontaneous circulation (ROSC) and survival in out-of-hospital cardiac arrest (OHCA) patients in a region with above-average survival rates.

**Design:**

Data on all OHCA patients in 2012 (non-TM group) were compared with those of all OHCA patients in 2018 (TM group). The association of the presence of a TM alert system with ROSC and survival was assessed with multivariate regression analyses.

**Results:**

TM responders reached 42 OHCA patients (15.9%) earlier than the first responders or ambulance. They connected 31 of these 42 OHCA patients (73.8%) to an AED before the ambulance arrived, leading to a higher percentage of AEDs being attached in 2018 compared to the 2012 non-TM group (55% vs 46%, *p* = 0.03). ROSC was achieved more often in the TM group (61.0% vs 29.4%, *p* < 0.01). Three-month and 1‑year survival did not differ significantly between the two groups (29.3% vs 24.3%, *p* = 0.19, and 25.9% vs 23.5%, *p* = 0.51). Multivariate regression analyses confirmed the positive association of ROSC with the TM alert system (odds ratio 1.49, 95% confidence interval 1.02‑2.19, *p* = 0.04).

**Conclusion:**

A TM alert system seems to improve the chain of survival; because TM responders reached patients early, AEDs were attached more often and more OHCA patients achieved ROSC. However, the introduction of a TM alert system was not associated with improved 3‑month or 1‑year survival in a region with above-average survival rates.

## What’s new?

- A text message (TM) alert system has benefits even in regions with already above-average survival of out-of-hospital cardiac arrest (OHCA) patients.
- Since the introduction of the TM alert system more automated external defibrillators have been attached to OHCA patients and return of spontaneous circulation has been achieved more often.
- No improvement in survival at 3 months or 1 year was found.

## Introduction

Survival rates following an out-of-hospital cardiac arrest (OHCA) are poor throughout the world [1]. To maximise survival in OHCA patients, a chain of survival was introduced at the 1992 National Conference on Cardiopulmonary Resuscitation and Emergency Cardiac Care [2]. The chain consists of four links: early access to the emergency medical services system, early cardiopulmonary resuscitation (CPR), early defibrillation and early advanced care.

For the ambulance services of the Hollands Midden region (RAVHM) in the mid-west of the Netherlands specific measures were introduced to optimise this chain of survival: police and fire departments were equipped with automated external defibrillators (AEDs) and officers were trained to perform adequate basic life support as first responders. Bystanders were supported by the dispatch centre in starting CPR. The ambulance staff perform CPR using a mechanical chest compression device, the Lund University Cardiopulmonary Assist System (LUCAS; Jolife AB, Lund, Sweden) and the Boussignac tube (Vygon, Ecouen, France) as the preferred assistive devices. These optimisations in the chain of survival have led to an improvement in the number of patients with return of spontaneous circulation (ROSC) on arrival at the emergency department of the hospital as well as in the 1‑year survival rate in the region served by the RAVHM as compared with the data for 2012 [3].

To further optimise the chain of survival in this region, a national text message (TM) alert system was implemented in 2016. This system alerts local lay responders to perform CPR or directs them to a nearby AED first [4]. Other studies have shown that a TM alert system can diminish the time to first shock and that this reduced delay is correlated with the availability of more TM responders and increasing density of AEDs [4, 5]. A Dutch study found higher survival rates in patients when a TM responder arrived at the scene [6]. A recent meta-analysis confirms the positive influence of a TM alert system, but also notes that no long-term survival data are available [7]. In addition, there are no outcome data of OHCA patients before and after the introduction of a TM system in a region in which survival is already above average, such as ours [3, 8].

This retrospective observational study compares the survival rate in OHCA patients in 2012, i.e. before the introduction of the TM alert system, with that of OHCA patients in 2018, 2 years after the introduction of this system. In addition, we investigated how often a TM responder reached OHCA patients earlier than regular emergency services and how often a TM responder attached an AED before the arrival of the emergency services.

## Methods

### Setting

The RAVHM provides ambulance services for approximately 775,000 inhabitants in the mid-west region of the Netherlands. Mean response time in 2012 was 9 min 59 s, in 2018 10 min 9 s (data from the validated time-keeping system OpenCare Ambu V.1.10/1.11; Centric, Gouda, The Netherlands).

When the dispatch centre is called for a (suspected) OHCA, two ambulances drive to the scene. First responders (i.e. policemen and firemen) are notified as well when they are in close proximity to the OHCA patient.

Since 2016 the dispatch centre of the study region has activated the TM alert system simultaneously with sending two ambulances: TM responders within a range of 750 m of the OHCA patient receive a text message on their cell phone directing them to the patient or to the location of the nearest AED first if available within a range of 500 m. All TM responders receive annual training from professional trainers in providing CPR. In 2018, there were 2.5 TM responders/km^2^ and 0.6 AEDs/km^2^.

### Study population

For the baseline population before the introduction of the TM alert system (the non-TM group) we used the study population of a previous observational prospective study [3]. These patients were included between November 2011 and 4 April 2013. For the population after the introduction of the TM alert system (the TM group), the electronic health records of the RAVHM for 2018 were searched for keywords possibly indicating a patient with OHCA.

Patients were included if they were older than 16 years and ambulance staff administered CPR and/or defibrillation. Patients were excluded if the national CPR protocol of the ambulance services was not started or stopped prematurely. Reasons were a do-not-resuscitate order (DNR) and conditions where resuscitation was found to be medically pointless (signs of biological death, lethal injury, a cardiac arrest period longer than 15 min without hypothermia, drowning or traumatic pulseless electrical activity).

### Data collection

Date collection complied with the Utstein template for uniform reporting of data from OHCA patients [9]. Patient characteristics and data concerning the CPR performed by the TM responders, the first responders and the ambulance staff were obtained from the electronic health records and Lifenet Code-Stat Reviewer 8.0 (Physio-Control, Redmond, WA, USA).

Follow-up data (i.e. survival after 3 months and 1 year) were collected for the OHCA patients that were brought to the Leiden University Medical Centre (LUMC). The LUMC is the only hospital in the region served by the RAVHM with intervention capacity (i.e. which performs acute primary coronary angioplasty) and therefore most OHCA patients are brought here. For some patients that were taken to other hospitals survival data were known because they were followed during a previous study [3].

The cardiology department of the LUMC refers eligible OHCA patients for cardiac rehabilitation to the Basalt Rehabilitation Centre in Leiden. In this rehabilitation centre screening for cognitive impairments is done in each OHCA patient with the option of following an additional cognitive rehabilitation programme for OHCA patients with post-anoxic encephalopathy. From the patient files of the Basalt Rehabilitation Centre, the Cerebral Performance Category (CPC) and modified Ranking Scale (mRS) scores were extracted. In addition, data on quality of life (QoL) at the end of cardiac rehabilitation were collected. QoL was measured with the Dutch translation of the MacNew Questionnaire, which was validated for a Dutch population [10].

The medical ethics committee of the LUMC approved the study protocol (G19.074). This study is registered in the Dutch Trial register (no. N7876). Because of the nature of OHCA and because patients received usual care, no informed consent was required.

### Statistical analysis

All data were anonymised when entered in a database. Statistical analysis was performed with SPSS version 25 (IBM Corp., Armonk, NY, USA). A two-tailed *p*-value of < 0.05 was considered to be statistical significant.

Baseline characteristics were compared using descriptive statistics using parametric and non-parametric tests when appropriate. Descriptive statistics were also used for the primary outcome measurements: the number of AEDs connected, ROSC at the emergency department, and 3‑month and 1‑year survival. In addition, a multivariate logistic regression model was created to further analyse the impact of the introduction of the TM alert system on the primary outcomes ROSC at the emergency department and 3‑month and 1‑year survival. Besides the TM alert system (reference: TM not present), age (in years), sex (reference: male), witness of OHCA (reference: witnessed OHCA), CPR before ambulance arrival (reference: CPR done), AED before ambulance arrival (reference: AED used), first monitored rhythm (reference: shockable), use of the LUCAS (reference: LUCAS used) and Boussignac tube (reference: Boussignac used) were used in the model. Spearman’s correlation coefficients were < 0.70 between all factors used in the model; therefore multicollinearity was excluded.

Complete data on survival were available for the LUMC. Therefore for this patient group data on survival, neurological outcome (i.e. CPC and mRS scores) and QoL were also analysed separately using parametric and non-parametric tests when appropriate.

## Results

**Fig. 1:**
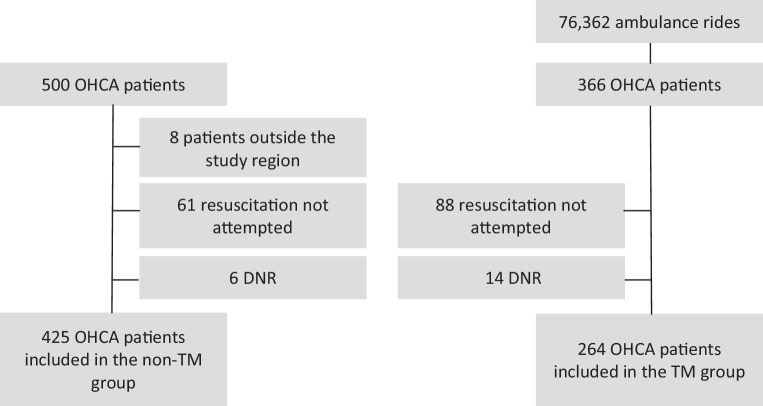
Flowchart. *OHCA* out-of-hospital cardiac arrest, *DNR* do-not-resuscitate order, *TM* text message

We included 425 of 500 OHCA patients in the 2012 non-TM group (85.0%) and 264 of 366 OHCA patients in the 2018 TM group (72.1%). A flowchart is given in Fig. 1. Age and sex were comparable between these two groups (Tab. 1). More patients in the non-TM group were at home when they had their OHCA compared to the TM group (67.3% vs 58.7%, *p* < 0.01).

**Table 1:** Characteristics of out-of-hospital cardiac arrest (*OHCA)* patients before the introduction of the text message (*TM*) alert system (non-TM group) and after the introduction of the system (TM group)

	Non-TM group	TM group	*p*
Resuscitations attempted	425	264	
Age (years), mean (range)	65.8 (16–100)	64.6 (18–95)	0.32
Male sex	299 (70.4%)	200 (75.8%)	0.12
*Location of OHCA*			< 0.01
– Home/residence	286 (67.3%)	155 (58.7%)	
– Street/public place	97 (22.8%)	91 (34.5%)	
– Industrial/workspace	10 (2.4%)	6 (2.3%)	
– Sport/recreational activity	13 (3.1%)	11 (4.2%)	
– Other	19 (4.5%)	1 (0.4%)	
Arrest witnessed/monitored	267 (63.7%)	165 (62.5%)	0.75
– Witnessed	221 (52.9%)	141 (53.4%)	
– Witnessed monitored	46 (10.8%)	24 (9.1%)	
Arrest not witnessed	152 (36.3%)	99 (37.5%)	
Missing	6	0	
CPR before ambulance arrival	308 (72.5%)	201 (76.1%)	0.29
AED before ambulance arrival	196 (46.1%)	144 (54.5%)	0.03
First monitored rhythm shockable	209 (49.2%)	132 (50.0%)	0.83
First monitored rhythm non-shockable	216 (50.8%)	132 (50.0%)	
Transported to hospital	332 (78.1%)	216 (81.8%)	0.24
– Leiden University Medical Centre	227 (53.4%)	149 (56.4%)	
– Other hospitals	105 (24.7%)	67 (25.4%)	
Pronounced dead at scene	93 (21.9%)	48 (18.2%)	
*Tools/devices used (multiple entry)*			
– LUCAS	327 (76.9%)	192 (72.7%)	0.21
– Endotracheal tube (Boussignac)	289 (68.0%)	131 (49.6%)	< 0.001
– Combined LUCAS/Boussignac	276 (64.9%)	121 (45.8%)	< 0.001

*CPR* cardiopulmonary resuscitation, *AED* automated external defibrillator, *LUCAS* Lund University Cardiopulmonary Assist System

TM responders reached 42 OHCA patients (15.9%) earlier than did the first responders or ambulance. In 31 of these 42 OHCA patients (73.8%) they attached an AED, leading to a significant higher percentage of AEDs connected in 2018 compared to the non-TM group in 2012 (55% vs 46%, *p* = 0.03). Although CPR was started more often before the ambulance arrived in the TM group (76.1%) than in the non-TM group (72.5%), this difference was not significant (*p* = 0.29).

ROSC was achieved in 210 patients in the non-TM group (49.4%) and in 161 patients in the TM group (61.0%, *p* < 0.01). Also in a multivariate regression correcting for confounders, the presence of a TM alert system was associated with higher odds of achieving ROSC (Tab. 2).

**Table 2:** Factors associated with return of spontaneous circulation (*ROSC*) at the emergency department, survival at 3 months and at 1 year in multivariate logistic regression analyses

		ROSC		3‑month survival			1‑year survival		
	Exp(β)	95% CI	*p*	Exp(β)	95% CI	*p*	Exp(β)	95% CI	*p*
TM alert system	1.49	1.02–2.19	0.04	1.17	0.74–1.85	0.50	1.00	0.63–1.61	0.99
Age	1.00	0.99–1.01	0.83	0.98	0.97–0.99	< 0.01	0.98	0.96–0.99	< 0.01
Male sex	1.48	0.98–2.23	0.07	1.47	0.89–2.41	0.13	1.63	0.98–2.71	0.06
Arrest witnessed	0.59	0.40–0.86	0.01	0.51	0.31–0.85	0.01	0.50	0.30‑0.84	0.01
CPR before ambulance arrival	1.27	0.76–2.12	0.37	1.36	0.74–2.49	0.32	1.25	0.67–2.34	0.48
AED before ambulance arrival	0.80	0.51–1.25	0.32	1.06	0.62–1.83	0.83	1.02	0.58–1.77	0.96
First monitored rhythm shockable	0.22	0.15–0.32	< 0.001	0.21	0.13–0.33	< 0.001	0.21	0.13–0.35	< 0.001
LUCAS used	10.05	5.34–18.91	< 0.001	3.33	1.91–5.83	< 0.001	3.52	2.00–6.20	< 0.001
Boussignac used	1.73	1.12–2.67	0.01	1.78	1.05–3.02	0.03	1.91	1.11–3.28	0.02

*CI* confidence interval, *TM* text message, *CPR* cardiopulmonary resuscitation, *AED* automated external defibrillator

**Table 3:** Outcomes of the out-of-hospital cardiac arrest patients brought to the Leiden University Medical Centre before the introduction of the text message (*TM*) alert system (non-TM group) and after the introduction of the system (TM group)

	Non-TM group		TM group		*p*
		*227*		*149*	
ROSC at the emergency department	227	147 (64.8%)	149	114 (76.5%)	0.02
Alive after 3 months	227	88 (38.8%)	147	63 (42.9%)	0.43
Alive after 1 year	227	85 (37.4%)	144	55 (38.2%)	0.89
Cerebral Performance Category score	40		32		0.44
– 1		29 (72.5%)		21 (65.6%)	
– 2		8 (20.0%)		10 (31.3%)	
– 3		3 (7.5%)		1 (3.1%)	
– 4 and 5		0 (0.0%)		0 (0.0%)	
Modified Rankin Scale score	40		32		0.98
– 0		8 (20.0%)		8 (25.0%)	
– 1		16 (40.0%)		12 (37.5%)	
– 2		11 (27.5%)		9 (28.1%)	
– 3		3 (7.5%)		2 (6.3%)	
– 4		2 (5.0%)		1 (3.1%)	
– 5 and 6		0 (0.0%)		0 (0%)	
Quality of life (mean ± SD)	31	147.1 ± 27.3	25	157.7 ± 28.3	0.17

*ROSC* recovery of spontaneous circulation, *SD* standard deviation

Survival rates decreased during follow-up, and consequently also the differences between the two groups. Of the non-TM group 88 patients (24.3%) were alive at 3 months and 85 (23.5%) at 1 year. Of the TM group 63 patients (29.3%) were alive at 3 months and 55 (25.9%) at 1 year (*p* = 0.19 and *p* = 0.51). Hence, the presence of a TM alert system was not associated with 3‑month or 1‑year survival in multivariate regression analyses, as shown in Tab. 2.

Complete data on survival of patients who were brought to the LUMC were available (Tab. 2). These survival data show the same trends. Of the OHCA patients who were alive at 3 months, 40 in the non-TM group (45.5%) and 32 in the TM group (50.8%) started a rehabilitation programme at the Basalt Rehabilitation Centre (*p* = 0.26). No differences were seen in neurological outcomes between the non-TM and the TM group (CPC *p* = 0.44, mRS *p* = 0.98). The majority of the patients in both groups had good outcomes.

## Discussion

This study demonstrates that, also in regions with above-average survival rates, the introduction of a TM alert system seems to further optimise the chain of survival: TM responders reached some of the patients earlier than the first responders or ambulance, an AED was connected more often and more OHCA patients achieved ROSC. However, there was no statistically significant improvement in 3‑month and 1‑year survival.

The number of TM responders reaching the OHCA patients earlier than first responders or the ambulance was relatively low compared to data in previous studies (15.9% vs 19%–95%) [7]. When they did arrive earlier, they were able to attach an AED in a high number of patients. In our study region the number of TM responders/km^2^ met the density of > 10 TM responders/km^2^ recommended by Stiegelis et al. [5], but the AED density was much lower (0.6 AEDs/km^2^) than the recommended 2 AEDs/km^2^. This might indicate that additional time is lost in reaching the nearest AED within a range of 500 m, during which the first responders or ambulance might already have arrived. A further increase in the density of AEDs and TM responders might further improve the benefit of the TM alert system in our region. However, the relatively low percentage of TM responders arriving earlier than first responders or the ambulance and the lack of a statistically significant effect on survival might also indicate that the chain of survival is near-optimal. Compared to the findings of previous studies [1, 8], survival rates in our region are high, and were so even before the introduction of the TM alert system.

No differences were seen in neurological outcomes, i.e. CPC and mRS scores, between the non-TM and TM group. The majority of both groups had good outcomes, as expected of a rehabilitation population. However, a rehabilitation population is not representative of the complete OHCA population: patients with severe cognitive dysfunction are not referred to a rehabilitation centre. However, the frequency of patients referred to the rehabilitation centre was not reduced, indirectly suggesting that neurological outcome was not worsened by the introduction of the TM alert system.

The most striking finding is the negative association between a witnessed arrest and a shockable rhythm and survival. In the literature there is abundant evidence that OHCA patients with a witnessed arrest and a shockable rhythm have a better chance of survival [8]. This difference in findings is explained by the selection of our population: patients in whom resuscitation was medically pointless were excluded from our study. The majority of these patients are those with a non-witnessed arrest and no shockable rhythm. However, because in both the non-TM and the TM group the same selection process took place and because the TM alert system is intended to contribute in the patients selected for the study, we expect that the selection of patients does not influence the results concerning the TM alert system.

Because this is a before and after intervention study, no randomisation took place and there might have been unmeasured confounders over time other than the introduction of the TM alert system. We have included known confounders in our regression analyses, such as bystander CPR. The same unchanged ambulance protocol was used in the two groups. In addition, there was no opportunity to select into or out of the intervention group: the TM alert system was already usual care. However, the Dutch guidelines on resuscitation from 2010 were updated in 2015, in which several small changes were made [11]. The main changes were: use of the telephone loudspeaker when a bystander alerts the emergency service, requirements of a DNR, a change in the CPR for children, a constant temperature between 32 °C and 36 °C during the 24 h after resuscitation, and a paragraph about rehabilitation. In addition, we cannot exclude the possibility that influential factors other than those we have measured might have changed between 2012 and 2018 in the two groups.

In conclusion, the TM alert system seems to contribute to optimisation of the chain of survival because it was associated with a higher ROSC rate, even in a region with already high survival rates and even though TM responders arrived earlier than first responders or the ambulance in only 15.9% of cases. However, after the acute phase, this contribution decreased and survival rates were not significantly different. More research is needed to establish whether a higher density of TM responders and perhaps also of AEDs would further increase the percentage of cases in which TM responders reach OHCA patients first and how to extend these improvements during the acute phase to improvements in the subacute and chronic phase.
